# Use of thiols and implications for the use of inhaled corticosteroids in the presence of oxidative stress in COPD

**DOI:** 10.1186/s12931-023-02500-8

**Published:** 2023-07-31

**Authors:** Mario Cazzola, Clive P. Page, Jadwiga A. Wedzicha, Bartolome R. Celli, Antonio Anzueto, Maria Gabriella Matera

**Affiliations:** 1 Chair of Respiratory Medicine, Department of Experimental Medicine, University of Rome Tor Vergata, Rome, Italy; 2 Sackler Institute of Pulmonary Pharmacology, Institute of Pharmaceutical Science, King’s College London, London, UK; 3 Respiratory Medicine Division, National Heart and Lung Institute, Imperial College London, London, UK; 4 Pulmonary and Critical Care Division, Brigham and Women’s Hospital, Harvard Medical School, Boston, MA USA; 5 Department of Pulmonary Medicine and Critical Care, University of Texas Health and South Texas Veterans Health Care System, San Antonio, TX USA; 6 Unit of Pharmacology, Department of Experimental Medicine, University of Campania Luigi Vanvitelli, Naples, Italy

**Keywords:** Antioxidant, Chronic obstructive pulmonary disease, Glutathione, Inhaled corticosteroids, Inflammation, Oxidative stress, Mucolytics, Thiol-based drugs

## Abstract

**Background:**

Oxidative stress and persistent airway inflammation are thought to be important contributors to the development of chronic obstructive pulmonary disease (COPD). This review summarizes the evidence for targeting oxidative stress and inflammation in patients with COPD with mucolytic/antioxidant thiols and inhaled corticosteroids (ICS), either alone or in combination.

**Main body:**

Oxidative stress is increased in COPD, particularly during acute exacerbations. It can be triggered by oxidant air pollutants and cigarette smoke and/or by endogenous reactive oxygen species (ROS) released from mitochondria and activated inflammatory, immune and epithelial cells in the airways, together with a reduction in endogenous antioxidants such as glutathione (GSH). Oxidative stress also drives chronic inflammation and disease progression in the airways by activating intracellular signalling pathways and the release of further inflammatory mediators. ICS are anti-inflammatory agents currently recommended for use with long-acting bronchodilators to prevent exacerbations in patients with moderate-to-severe COPD, especially those with eosinophilic airway inflammation. However, corticosteroids can also increase oxidative stress, which may in turn reduce corticosteroid sensitivity in patients by several mechanisms. Thiol-based agents such as erdosteine, N-acetyl L-cysteine (NAC) and S-carboxymethylcysteine (S-CMC) are mucolytic agents that also act as antioxidants. These agents may reduce oxidative stress directly through the free sulfhydryl groups, serving as a source of reducing equivalents and indirectly though intracellular GSH replenishment. Few studies have compared the effects of corticosteroids and thiol agents on oxidative stress, but there is some evidence for greater antioxidant effects when they are administered together. The current Global Initiative for Chronic Obstructive Lung Disease (GOLD) report supports treatment with antioxidants (erdosteine, NAC, S-CMC) in addition to standard-of-care therapy as they have been demonstrated to reduce COPD exacerbations. However, such studies have demonstrated that NAC and S-CMC reduced the exacerbation risk only in patients not treated with ICS, whereas erdosteine reduced COPD exacerbations irrespective of concomitant ICS use suggesting that erdosteine has additional pharmacological actions to ICS.

**Conclusions:**

Further clinical trials of antioxidant agents with and without ICS are needed to better understand the place of thiol-based drugs in the treatment of patients with COPD.

## Inflammation and oxidative stress

The pathogenesis of chronic obstructive pulmonary disease (COPD) is complex and not yet fully understood but is characterised by the presence of both oxidative stress and persistent airway inflammation. Typically, there is an abnormal inflammatory immune response of the lungs to air pollutants, such as ozone, nitrogen dioxide, combustion particulates and gases, usually from cigarette smoke and oxidant air pollution [1]. The inflammation is usually associated with increased numbers of neutrophils, activated macrophages and activated T-lymphocytes in the lungs [2]. However, 20–40% of patients with COPD have an eosinophil-rich infiltrate, whilst other subgroups demonstrate a neutrophilic inflammation that is combined to varying degrees with eosinophilic inflammation [3]. This persistent inflammation leads to progressive and irreversible airflow obstruction and the recurrent acute episodes of worsening (exacerbations) that characterise COPD [1, 2].

In the recent Rome Proposal, a COPD exacerbation was described as an event characterised by an acute burst of airways inflammation due to stimuli (e.g., bacteria, viruses, or environmental pollutants) that is coupled with worsening of existing airflow limitation, leading to worsening symptoms (dyspnoea and/or cough and sputum), which may be accompanied by tachypnea and/or tachycardia [4]. For most patients, the time from onset of worsening respiratory symptoms to a full exacerbation of COPD is within 5 days [4, 5]. The airway and systemic inflammation are increased during exacerbations, and the systemic inflammatory component may be linked with the co-morbidities associated with the disease [6, 7].

The presence of inflammation in the lungs of patients with COPD is intimately linked to oxidative stress [8, 9], which is an imbalance between oxidant production and antioxidant defences in favour of oxidants. Under normal physiological conditions, oxidants generated include free radicals, such as superoxide anions (O_2_^−^), which can lead to the formation of other reactive oxygen species (ROS), including hydroxyl radicals (OH^−^) and hydrogen peroxide (H_2_O_2_); ROS are cleared by endogenous antioxidants [10]. The balance between ROS production and antioxidant defences determined the degree of oxidative stress [11].

Oxidative stress is markedly increased in patients with COPD, especially during acute exacerbations, and contributes to the pathology of the disease [12, 13]. It can be caused by either exogenous or endogenous oxidants, and inflammatory and structural cells within the lungs can be both the target and source of oxidants [13].

**Fig. 1:**
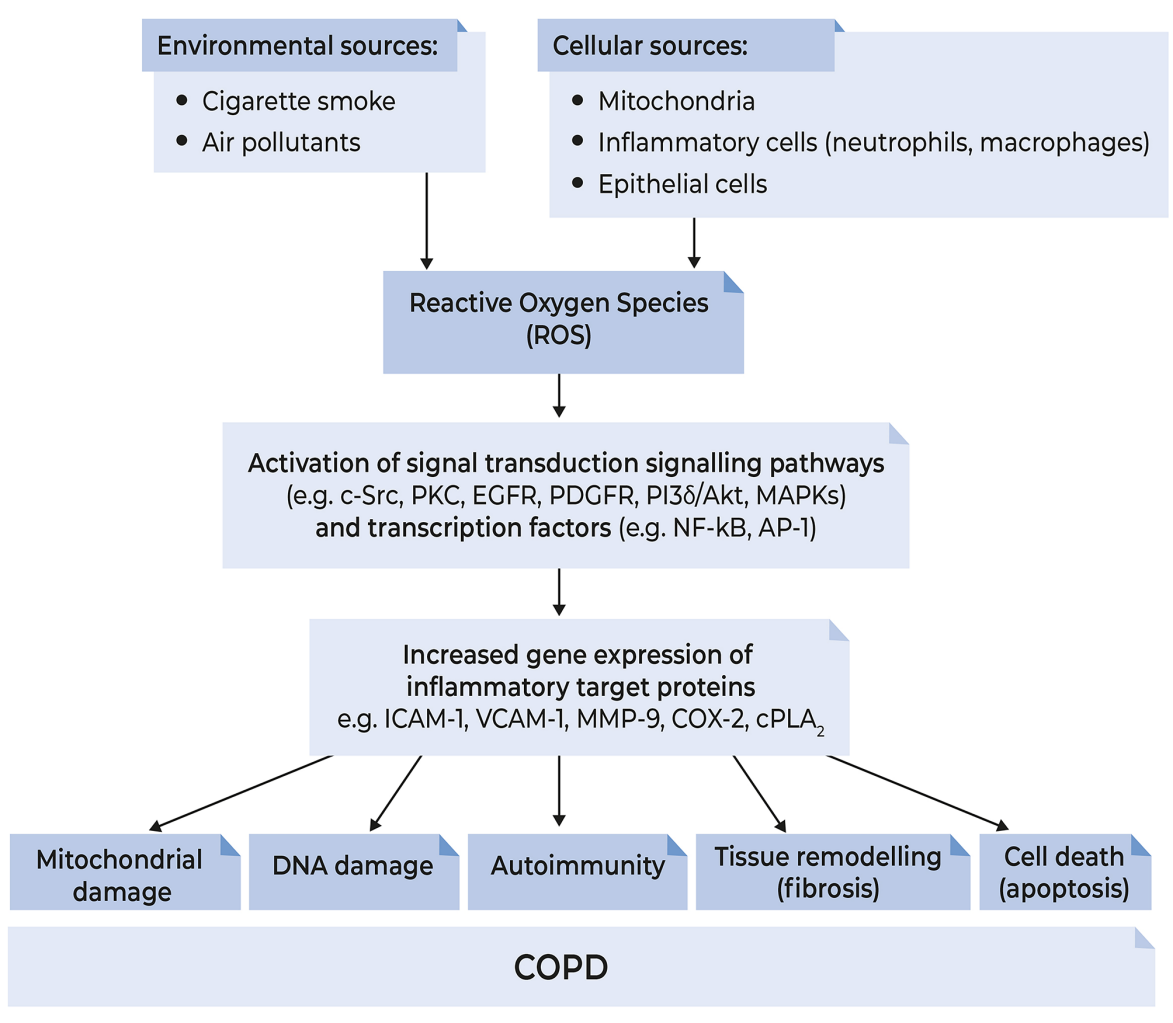
Events caused by oxidative stress and reactive oxygen species (ROS) production in patients with COPD. Oxidative stress occurs in response to inhaled oxidants from environmental sources and excess production of cellular ROS, leading to an imbalance between oxidants and antioxidants in favour of oxidants. The ROS cause lipid peroxidation and oxidative damage to proteins and DNA, as well as activation of cells and signal transduction pathways that trigger inflammatory mediator release and protease activation, which sustain the oxidative stress. *AP-1* activated protein-1, *COPD* chronic obstructive pulmonary disease, *COX-2* cyclooxygenase-2, *cPLA*_*2*_ cytosolic phospholipase A2, *c-Src* cellular Src (a non-receptor tyrosine kinase protein), *DNA* deoxyribonucleic acid, *EGFR* epidermal growth factor receptor, *ICAM-1* intracellular adhesion molecule-1, *MAPK* mitogen-activated protein kinase, *MMP-9* matrix metalloproteinase 9, *NF-κB* nuclear factor-κappaB, *PDGFR* platelet-derived growth factor receptor, *PI3Kδ* phosphoinositide-3-kinase-delta, *PKC* protein kinase C, *ROS* reactive oxygen species, *VCAM-1* vascular cell adhesion molecule 1

ROS are signalling molecules that influence many processes [10], and can initiate inflammatory responses through the activation of various signal transduction pathways and transcription factors, leading to increased gene expression of inflammatory target proteins [14] (Fig. 1). The degree to which a given transcription factor is activated is highly dependent on the nature and duration of the stress, as well as the cell type [11]. Enhanced expression of proinflammatory mediators, including cytokines and peroxidation products of arachidonic acid (leukotrienes, prostanoids and isoprostanes), also occurs. Neutrophils and other inflammatory cells are attracted into the lungs and the resulting inflammatory response (pulmonary and systemic) and cell apoptosis are the final products of this cascade of reactions [14].

ROS cause tissue damage via lipid peroxidation and oxidation of both proteins and carbohydrates, leading to carbonyl stress, with accumulation of reactive carbonyls and subsequent protein carbonylation [15]. In turn, oxidative stress elicits nonenzymatic post-translational alterations resulting in dysfunctional proteins, the formation of danger-associated molecular patterns (DAMPs) and neo-autoantigens [15]. However, sophisticated adaptive enzymatic mechanisms including superoxide dismutase (SOD), catalase and glutathione peroxidase (GPX), and nonenzymatic antioxidant defence systems such as glutathione (GSH) and vitamins (A, C, E) have evolved to metabolise ROS into less reactive forms to maintain physiological homeostasis and protect cells against oxidant stress [11, 16].

GSH is a low-molecular-weight thiol present in cells at millimolar concentrations. It plays important roles in cellular defence against oxidant stimulation, in redox regulation of protein thiols and in maintaining redox homeostasis, which is essential for the proper functioning of cellular processes including apoptosis [17]. GSH acts as an antioxidant either directly by interacting with ROS/reactive nitrogen species and electrophiles, or by operating as a reductant substrate for other enzymatic (e.g., GPX) and non-enzymatic (e.g., vitamin C) antioxidants [18].

**Fig. 2:**
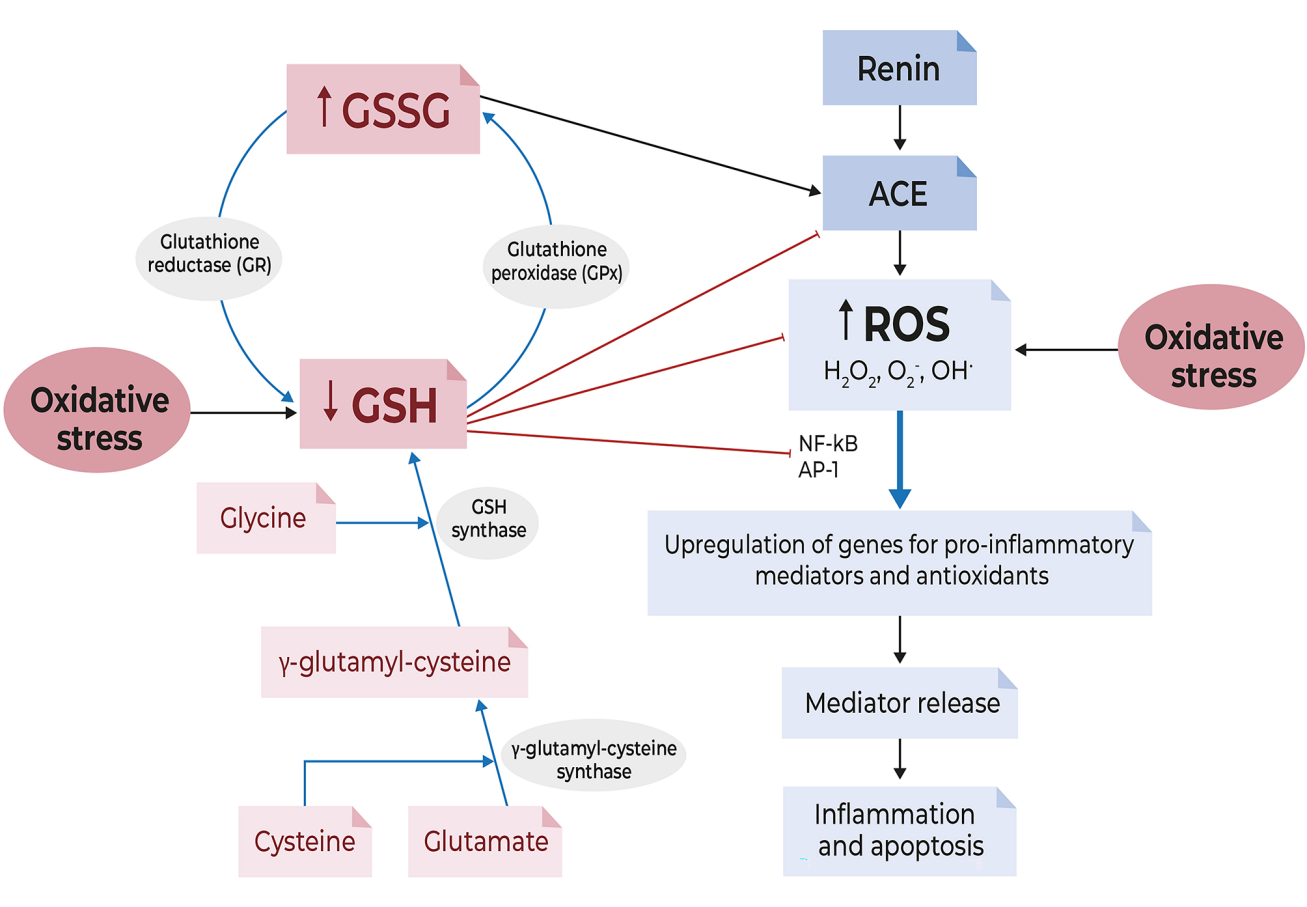
Role of glutathione in oxidative stress. During oxidative stress there is an increase in oxidants (ROS) and a decrease in antioxidants such as GSH (the reduced form of glutathione). ROS activate transcription factors nuclear factor kappaB (NF-κB) and activator-protein-1 (AP-1), signal transduction pathways and release of pro-inflammatory mediators. GSH inhibits angiotensin-converting enzyme (ACE) activity, neutralizes (reduces) ROS and decreases activation of NF-κB and AP-1 [red lines]. *ROS* reactive oxygen species, *GSSG* oxidized glutathione

Antioxidant capacity in the lung is substantially reduced and oxidative stress can persist long after the cessation of cigarette smoking or an acute exacerbation of COPD owing to continued production of ROS from endogenous sources [9, 12]. The loss of antioxidant capacity during oxidative stress is mainly due to a depletion of GSH and/or its precursor, cysteine [17]. GSH is synthesised in a two-step enzymatic process (Fig. 2), where cysteine is the limiting amino acid [19]. The presence of ROS promotes oxidation of cysteine to its disulphide form (i.e., cystine) and further depletes the pool of cysteine available [20]. Diminished GSH levels elevate cellular vulnerability towards oxidative stress and contributes to pro-inflammatory cytokine release, free radical formation, inhibition of macrophage and natural killer cell functionality along with disease susceptibility and progression [21] (Fig. 2). Evidence indicates that low intracellular thiol/GSH levels promote nuclear factor (NF)-κB activation, whereas high intracellular thiol/GSH levels degrade NF-κB [22, 23].

Studies in COPD patients have shown that GSH levels are increased in the bronchoalveolar lavage (BAL) fluid of smokers versus non-smokers and are reduced during severe exacerbations compared with stable COPD [24].

To achieve therapeutic levels, high doses of GSH must be administered because it has a short half-life in blood plasma. Furthermore, GSH cannot cross cell membranes, but first needs to be broken down into its constituent amino acids and then re-synthesised inside the cell. To overcome these limitations of GSH, pro-GSH molecules could be used to restore or increase GSH levels [25].

### Inhaled corticosteroids and oxidative stress

Suppression of the inflammatory response is a valid therapeutic goal for patients with COPD that aims to improve symptoms such as cough and mucus secretion, improve health status and reduce exacerbations [26].

Corticosteroids are currently the main class of anti-inflammatory drugs used in the treatment of COPD to prevent exacerbations. Although these drugs are known to effectively suppress airway inflammation in subjects with asthma, their effect on the inflammation in COPD remains unclear [27]. The value of ICS in the treatment of COPD and understanding of which patients may benefit from ICS therapy is becoming clearer, but a consensus is still lacking [28]. The current GOLD report recommends the use of ICS in patients with severe impairment and at high risk of exacerbation [29]. However, there is evidence both in favour and against the efficacy of corticosteroids in treating the inflammatory processes in COPD [30].

**Fig. 3:**
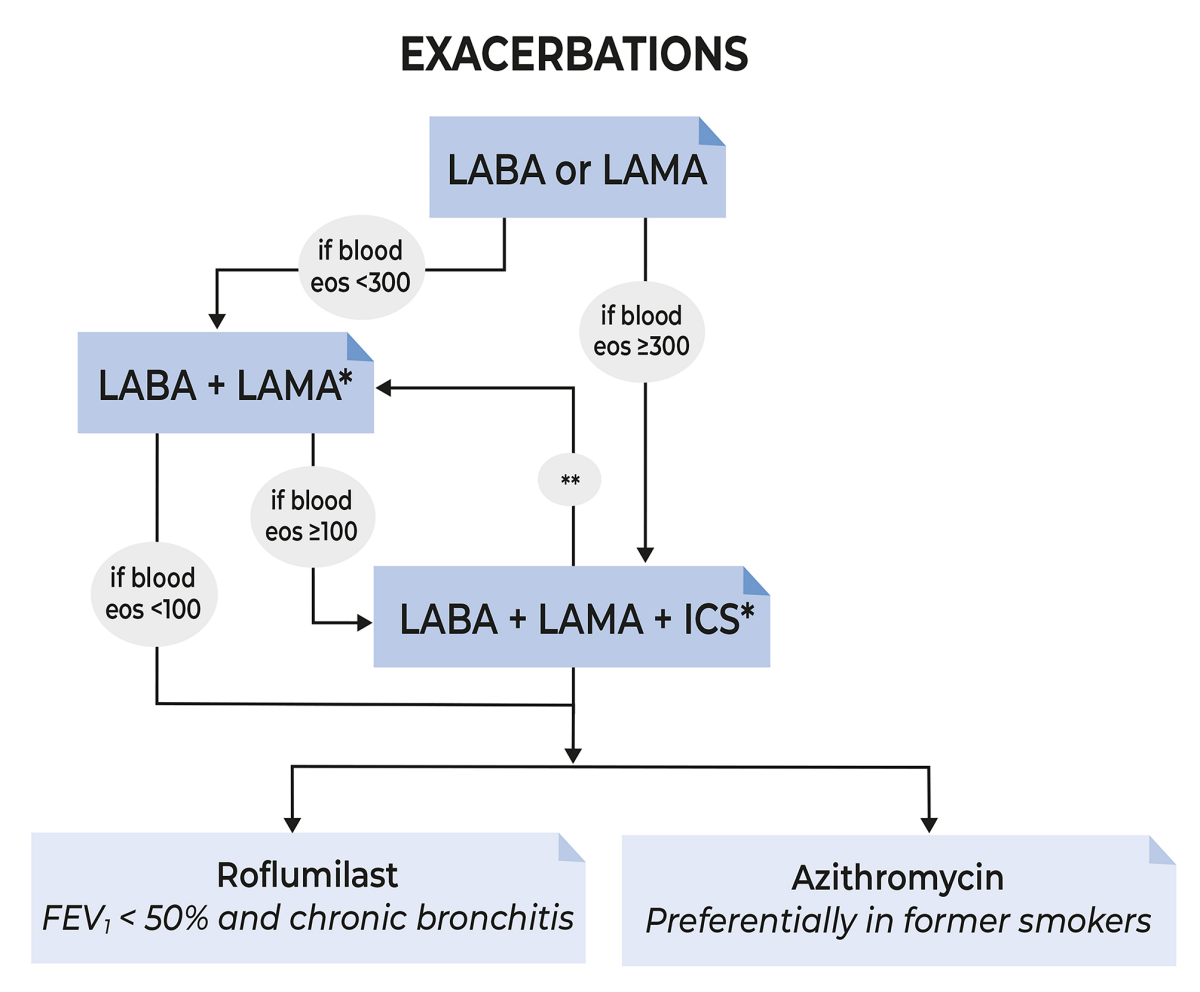
Use of ICS in patients with COPD exacerbations according to recommendations in GOLD Report 2023 [29]. *Single inhaler therapy may be more convenient and effective than multiple inhalers. **Consider de-escalation of ICS if pneumonia or other considerable side-effects. In case of blood eos ≥ 300 cells/µl de-escalation is more likely to be associated with the development of exacerbations. *Eos* blood eosinophil count, *ICS* inhaled corticosteroid, *LABA* long-acting beta2-agonist, *LAMA* long-acting muscarinic antagonist

It has been suggested that the heterogeneity of patients with COPD necessitates different treatment strategies for different subgroups of patients [31]. In the past, ICS have been considered more effective in frequent exacerbators or those with an overlap between COPD and asthma [28]. In the recent 2023 GOLD report [29], emphasis is given to the use of ICS combined with a long-acting beta-agonist (LABA) and a long-acting muscarinic-antagonist (LAMA) in individuals with a blood eosinophil count ≥ 300 cells/µL (Fig. 3). GOLD 2023 also recommends considering the addition of ICS in patients who experience an exacerbation despite treatment with LABA + LAMA when the blood eosinophil count is > 100 cells/µl. These recommendations are based on the findings from randomised controlled trials (e.g., ETHOS, IMPACT) and systematic reviews and meta-analyses that showed the effectiveness of ICS in preventing COPD exacerbations in COPD patients with blood eosinophil counts > 100 cells/µl [28, 32–34].

Dual bronchodilation therapy plays a fundamental role in the treatment of the general COPD population, while ICS-containing therapy provides clinical benefit in the subgroup of patients with significant blood eosinophilia or a high-risk for exacerbation [35]. However, neutrophilic inflammation is the dominant “endotype” in COPD patients [36] and the neutrophilic inflammatory pathway responds poorly to corticosteroids [37]. Indeed, corticosteroids prolong neutrophil survival by inhibiting their apoptosis and even high doses of oral corticosteroids or ICS fail to reduce the sputum neutrophilia [9].

There is evidence that oxidative stress may reduce corticosteroid sensitivity in COPD by mechanisms involving phosphorylation of the glucocorticoid receptor (GR) as well as phosphorylation and inactivation of histone deacetylase-2 (HDAC-2) [38–41]. A poor clinical response to ICS therapy in COPD patients has been associated with reduced GR expression on airway neutrophils, but not on blood neutrophils [42]. An in vitro study of human bronchial epithelial cells from patients with COPD or asthma showed that increased oxidant burden may lead to reduced corticosteroid responsiveness in airway epithelium, with important consequences for the integrity of the epithelial barrier and associated production of pro-inflammatory cytokines [43]. It has been suggested that exogenous oxidative stress reduces the expression of nuclear RanGTPase-activating protein, which is essential for the nuclear import of cargo proteins and re-localisation of importin-β, resulting in impaired nuclear import, and thereby the reduced GR function and corticosteroid responsiveness seen in patients with COPD [44]. Cysteine oxidation promotes a post-translational modification of the GR that impairs its nuclear expression and binding to the glucocorticoid consensus and modifies the GR protein by decreasing available sulfhydryl groups and decreasing nuclear GR expression and activity [20]. All this is associated with decreased responsiveness to corticosteroids.

Several preclinical studies have shown that corticosteroids retain some activity in the presence of oxidative stress. In a study from 1997, dexamethasone administered subcutaneously displayed antioxidant properties in the lungs of adult rats, with an increase in antioxidant enzymes (catalase, GPX and SOD) that was achieved by enzyme induction rather than through an activation process [45]. Also, prednisolone and dexamethasone directly inhibited the production of intracellular ROS by human platelets [46]. Furthermore, in a rat model of collagen-induced rheumatoid arthritis, the glucocorticoid deflazacort not only significantly decreased the level of articular elastase, nitric oxide and lipid peroxidation, but also significantly increased the activity of catalase, SOD and GSH [47]. These effects may contribute to the anti-inflammatory effects of corticosteroids.

Paradoxically, corticosteroids can also increase oxidative stress. Although acute administration of methylprednisolone to rats increased antioxidant levels in lung tissue, suggesting potential benefits of corticosteroid use during acute events, chronic administration of methylprednisolone increased lung lipid peroxidation levels, indicating an increased risk of oxidative lung injury [48]. There are also other reports of oxidative injury caused by increased lipid peroxidation and inhibition of key antioxidant enzymes in response to high glucocorticoid exposure in hippocampal slice cultures, vascular endothelial cells, thymocytes and myeloma cells [49–52]. A meta-analysis of glucocorticoid-induced physiological stress studies found that corticosteroids increase oxidative stress, most notably after 3 weeks of administration [53].

### Thiols and oxidative stress

**Fig. 4:**
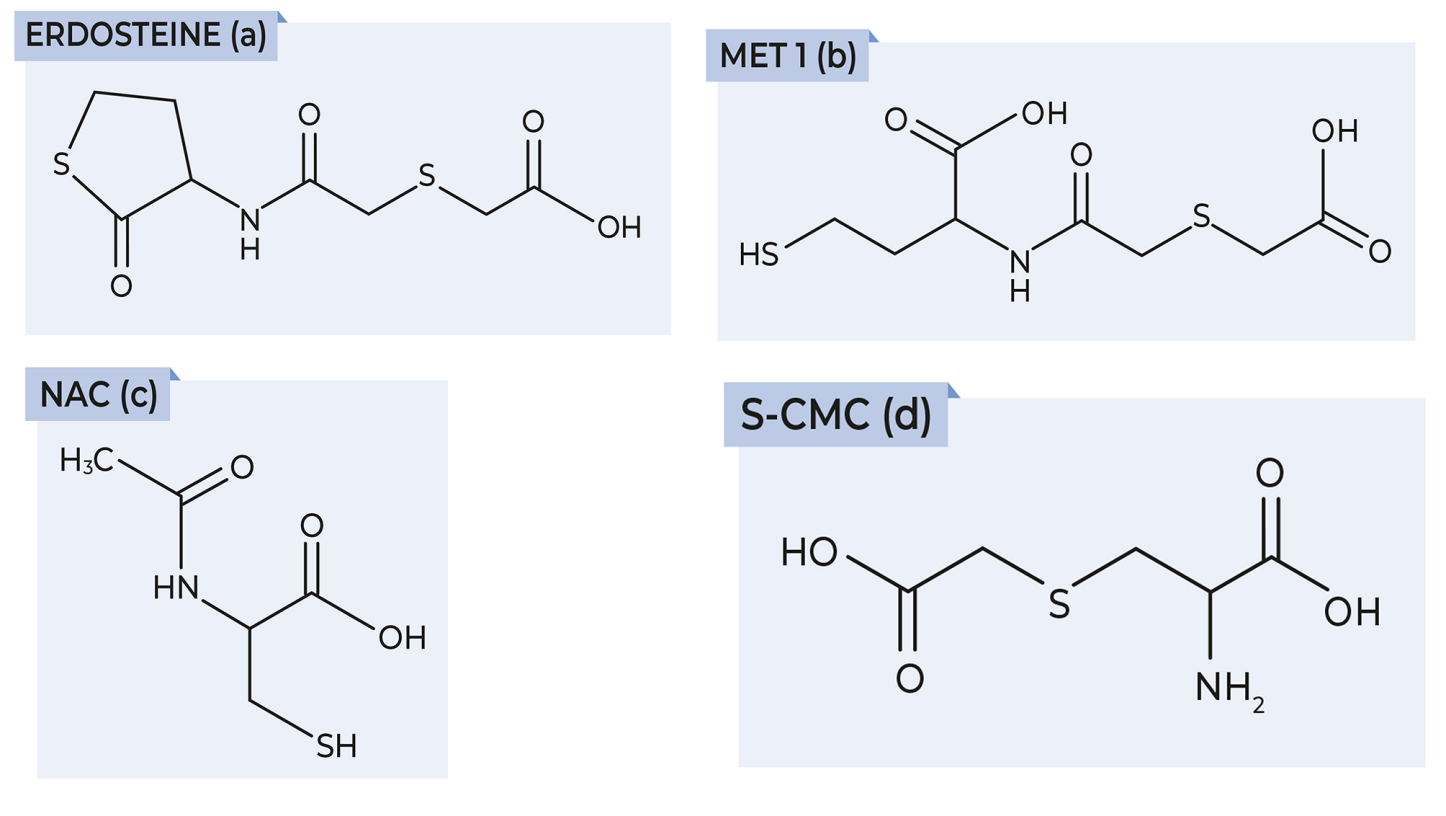
Chemical structures of thiol-based antioxidants

Drugs containing the thiol moiety (–SH) or that are metabolised to thiol-containing species, such as N-(carboxymethylthioacetyl)-homocysteine thiolactone (erdosteine), N-acetyl-L-cysteine (NAC) and S-carboxymethylcysteine (S-CMC) (Fig. 4), have been shown to reduce oxidative stress by direct antioxidant effects due to the free sulphydryl (–SH) groups serving as a source of reducing equivalents, as well as by indirect antioxidant effects through the replenishment of intracellular GSH levels [18].

Erdosteine (Fig. 4a) is a prodrug that is metabolised to the ring-opening compound Metabolite 1 (Met 1, N-thiodiglycolylhomocysteine) after first-pass metabolism. Met 1 (Fig. 4b) exerts an antioxidant effect on neutrophils and eosinophils because of the scavenging effect of its pharmacologically active –SH group, which is a good electron donor [54]. Experimental evidence in animals supports the protective effect of erdosteine against various types of oxidative stress-induced lung tissue injury [55–57]. For example, erdosteine protected rats against ischaemia-reperfusion-induced lung injury, as shown by reduced malondialdehyde (MDA) levels and myeloperoxidase (MPO) activity in lung tissue and decreased neutrophil counts in BAL, compared to the control group [58]. In bleomycin-induced lung fibrosis in rats, erdosteine prevented the increase in MDA, nitrate/nitrite and hydroxyproline levels and inhibited the decrease in GSH and SOD levels in lung tissue [59]. Thus, erdosteine not only prevents the accumulation of free oxygen radicals when their production is accelerated, but also increases antioxidant cellular protective mechanisms [60]. Furthermore, erdosteine is effective in preventing H_2_O_2_-induced oxidative stress and DNA damage in lung epithelial cells through the scavenging of intracellular ROS [61].

Studies in COPD patients provide supportive clinical evidence that erdosteine is effective against oxidative stress [62]. Erdosteine treatment was associated with decreases in plasma ROS and 8-isoprostane levels in COPD patients who were current smokers [63, 64], those undergoing acute exacerbations of COPD [65], and in patients with exercise-induced oxidative stress [66].

NAC (Fig. 4c) is also able to scavenge oxidants directly through its thiol moiety and act as a source of cysteine for the biosynthesis of GSH [67]. However, it is likely that NAC influences oxidative stress indirectly through the replenishment of intracellular GSH [17, 18] because the rate constants for its reaction with O_2_^–^ and H_2_O_2_ are too low to make a significant contribution to oxidant scavenging [68]. Indirect antioxidant effects of NAC were demonstrated in a human ex *vivo* model of COPD exacerbation induced by lipopolysaccharide (LPS) [69, 70], and confirmed in patients with COPD who showed increased GSH levels in plasma and BAL after NAC treatment [71].

S-CMC (Fig. 4d) is a thiol derivative of L-cysteine that differs in structure and mechanism of action from NAC and erdosteine because it does not have a free thiol (SH) group [72]. S-CMC has a thioether structure and in vitro cell-based studies suggest it may exert antioxidant effects by directly scavenging ROS [73] and preventing oxidative stress-induced GSH efflux from cells [74]. There is only limited evidence for antioxidant activity of S-CMC in COPD patients [72], including reduced concentrations of exhaled 8-isoprostane (a marker of lipid peroxidation) in COPD patients treated with S-CMC [75].

Several studies have compared the antioxidant activity of these different thiol agents. In an in vitro study comparing the inhibitory effects of erdosteine (Met 1), S-CMC and NAC on ROS produced by stimulated rat neutrophils, guinea pig eosinophils and human neutrophils, the effects of S-CMC and NAC were weaker than those of erdosteine [76]. In a clinical study comparing erdosteine and NAC treatment in patients with chronic bronchitis, GSH levels in plasma and BAL were significantly higher after erdosteine treatment, compared to NAC [77]. In a randomised, placebo-controlled study comparing the effects of erdosteine and NAC treatment for 10 days in 30 patients with mild-to-moderate COPD who were current smokers, both erdosteine and NAC caused significant reductions in ROS blood levels, but only erdosteine lowered 8-isoprostane levels and restored the short-term FEV_1_ response to salbutamol [64]. The antioxidant activity of erdosteine in COPD patients has been shown to be dose-dependent [65].

### Comparing the antioxidant effects of corticosteroids and thiols

Few experimental studies or clinical trials in COPD patients have directly compared the effects of corticosteroids versus thiol agents on oxidative stress.

In an in vitro study of bronchial epithelial cells exposed to the oxidant activities of cigarette smoke extracts, S-CMC, but not fluticasone propionate, reduced ROS production, increased GSH, heme oxygenase-1 and HDAC-2 nuclear expression/activity [78]. In contrast, in an ex vivo experimental study of acute lung inflammation in mice exposed to aerosol endotoxin (LPS), there was a significant reduction of oxygen radicals in neutrophils obtained from BAL fluid with dexamethasone but not NAC, demonstrating a differential anti-oxidative effect of the two drugs [79]. In a mouse model of chlorine-induced acute lung injury, neither dexamethasone nor NAC reduced airway hyperreactivity or decreased inflammatory cell influx in BAL [80]. However, in another model of acute lung injury due to pulmonary contusion in rats, early administration of dexamethasone or NAC decreased MDA and other ROS in lung tissue, but only dexamethasone significantly decreased neutrophils in BAL fluid [81].

Treatment of COPD patients with oral corticosteroids, even for a short time, resulted in lower plasma MDA levels, an effect attributable to inhibition of lipid peroxidation, while NAC showed no significant effect on oxidant/antioxidant status, as measured by MDA or SOD levels [82].

In a study examining whether ICS and NAC have any effect on oxidative stress markers in patients with stable COPD, fluticasone propionate administered for 10 weeks led to a small improvement from baseline in the systemic redox balance by increasing GPX activity, whereas 10 weeks of NAC tended to decrease interleukin (IL)-8 levels in sputum, but did not affect trolox equivalent antioxidant capacity (TEAC), which reflects antioxidant activity against ROS and reactive nitrogen species [83].

### Combining the antioxidant effects of corticosteroids and thiols

The different antioxidant mechanisms activated by corticosteroids and thiols suggest that a combination of these two classes of drug may be useful in counteracting oxidative stress. A synergistic antioxidant effect was observed when budesonide and erdosteine were administered together to stimulated human neutrophils undergoing respiratory bursts [84]. NAC increased glucocorticoid sensitivity in a mouse model of steroid-resistant asthma [85] and mitigated the oxidative stress damaging effects of dexamethasone [86].

A small study in COPD patients compared the effects on oxidative stress of 6-months treatment with unapproved high-dose NAC (1200 mg/day) alone or in combination with an ICS [87]. MDA plasma levels from baseline to 6 months and the authors concluded that the combination of NAC and ICS, but not NAC alone, reduced the oxidant burden in airways.

In cellular and animal models of steroid insensitivity mediated by oxidative stress, S-CMC restored steroid sensitivity by increasing HDAC-2 expression/activity through elevating GSH and SOD levels, reducing oxidative stress, enhancing HDAC-2 recruitment and reducing H4 acetylation in the IL-8 promoter region, and eventually alleviating inflammation and lung damage [88, 89].

S-CMC alone and combined with beclomethasone dipropionate can counteract the effects of cigarette smoke exposure on chromatin remodelling and increased pro-inflammatory responses in human bronchial epithelial cells in vitro [90].

### Expert opinion

ICS are widely prescribed in COPD, either alone or in combination with other drugs; reports in the literature estimate that up to 86% of COPD patients are treated with ICS, regardless of COPD severity and exacerbation risk [91]. The high percentage of COPD patients being prescribed ICS-based maintenance therapy was confirmed in a recent Delphi survey of COPD management across six European countries [92].

As oxidative stress is increased in COPD patients, particularly during acute exacerbations [9], and NAC, S-CMC and erdosteine have been shown to reduce the risk of COPD exacerbations [93–95], it seems logical that antioxidants may provide additional benefit to treatment with an ICS. It is possible that corticosteroids, which work in part by reducing the expression of pro-inflammatory genes [40], could promote a faster and more efficient protection against oxidative stress when used in combination with a thiol agent. Moreover, there may be certain subgroups of COPD patients who have more pronounced oxidative stress and who, therefore, would benefit from a more effective pharmacological approach with an antioxidant instead of an ICS. For example, a study in COPD patients with metabolic syndrome showed they had higher levels of advanced oxidation protein products, reflecting a greater degree of oxidative stress, than those without metabolic syndrome [96]. In such patients, a thiol agent may be effective alone or in combination with a low dose of corticosteroid. Indeed, it was suggested in the late 1990s that antioxidant treatment might be relatively more effective among those COPD patients who respond less well to ICS (heavy smokers with largely irreversible airflow obstruction) [97].

In 2019, the GOLD international guidelines recommended the use of oral antioxidant mucolytic agents, such as NAC, S-CMC or erdosteine, in patients with COPD who were not receiving ICS, as these agents may reduce exacerbations and improve patient health status [98]. This recommendation, which has since been updated in the GOLD 2023 Report [29], was based primarily on the evidence available for each mucolytic agent at that time.

The BRONCUS (Bronchitis Randomized On NAC Cost Utility Study) study indicated the possible benefit of the approved standard dose of NAC (600 mg/day) in reducing exacerbations in patients with moderate-to-severe COPD, but this effect may be limited to patients not treated with ICS [99]. In contrast, the placebo-controlled PANTHEON (Placebo-controlled study on efficAcy and safety of N-acetylcysTeine High dose in Exacerbations of chronic Obstructive pulmoNary disease) study, conducted in Chinese patients with moderate-to-severe COPD, concluded that the reduction of acute exacerbations during NAC treatment, administered at an unapproved high dose (1200 mg/day), was independent of concomitant ICS use [93]. However, a post-hoc analysis of this trial showed that, in general, the efficacy of NAC was highest in patients not receiving ICS as background COPD medication [100].

There is only limited clinical evidence of the long-term effects of S-CMC in COPD patients receiving concomitant ICS. In the PEACE study, a randomised, placebo-controlled study in Chinese patients with COPD, there was a non-significant interaction between the preventive effects of S-CMC on acute exacerbations and concomitant ICS use, but > 50% of patients had GOLD stage III or IV COPD and only 16.7% of study participants were receiving ICS and at a low dose [94]. This contrasts with the reports discussed earlier that there is widespread use of ICS in COPD patients (up to 86%) and, thus, the data generated in this study may not be representative of the COPD population using ICS in normal clinical practice.

The RESTORE (Reducing Exacerbations and Symptoms by Treatment with Oral Erdosteine in COPD) placebo-controlled study demonstrated that erdosteine administered twice daily at the approved dose (300 mg twice daily) and added to usual maintenance therapy for 12 months, reduced the overall exacerbation rate in patients with moderate or severe COPD and a history of exacerbations in the previous year, many of whom were already receiving ICS [95]. The results on exacerbation outcomes over the 12-month study period showed that the efficacy of erdosteine did not differ between ICS users and non-users in the total RESTORE population (Table 1). As more than half of all COPD patients in the community have moderate COPD, a post-hoc analysis of the RESTORE study was performed to investigate whether the subgroup of COPD patients with moderate airflow limitation (57% of the total study population) would benefit specifically from erdosteine treatment [101]. The exacerbation data for the moderate COPD subgroup not only showed a significant reduction in exacerbation rate compared to placebo, but also there were no differences in exacerbation frequency and duration between ICS users and non-users. Furthermore, the time to first exacerbation was prolonged in the erdosteine group and this was not influenced by the concomitant use of ICS (Table 1).

**Table 1:** Exacerbation data by treatment group and ICS users/non-users in RESTORE study [95, 101]

	Erdosteine	Placebo	Difference vs. placebo (%)	p-value^b^
**Exacerbation rate, per patient per year**				
*All RESTORE patients* (N = 445)	N = 215	N = 230		
All patients	0.91	1.13	-19.4	0.01
ICS users^a^ (N = 338)	0.93	1.16	-19.5	0.02
ICS non-users (N = 107)	0.89	1.10	-19.3	0.01
*Moderate COPD subgroup* (N = 254)	N = 126	N = 128		
All patients in subgroup	0.27	0.51	−47.2	0.003
ICS users^a^ (N = 135)	0.30	0.54	−44.4	0.002
ICS non-users (N = 119)	0.26	0.49	−46.9	0.003
**Time to first exacerbation in days, mean (SD)**				
*RESTORE patients* ^*c*^ *(N = 224)*	171 (27)	164 (28)	+ 3.6	0.020
ICS users (N = 63)	173 (29)	166 (27)	+ 4.0	0.028
ICS non-users (N = 161)	171 (24)	165 (28)	+ 3.5	0.048
*Moderate COPD subgroup (N = 163)*	182 (19)	169 (25)	+ 7.7	< 0.001
ICS users (N = 30)	180 (23)	168 (24)	+ 7.1	< 0.001
ICS non-users (N = 133)	185 (18)	170 (23)	+ 8.8	< 0.001
**Exacerbation duration in days, mean (SD)**				
*All RESTORE patients (N = 445)*				
All exacerbations	9.5 (7.2)	12.6 (9.7)	-24.6 (5.3)	0.023
Mild exacerbations	8.4 (5.2)	10.4 (8.2)	-19.2 (4.9)	0.039
Moderate-to-severe exacerbations	11.1 (8.9)	14.1 (10.8)	-21.3 (5.2)	0.041
*Moderate COPD subgroup (N = 254)*				
All exacerbations	9.1 (7.4)	12.3 (9.6)	−26.0 (5.6)	0.022
Mild exacerbations	7.7 (4.9)	9.8 (8.0)	−21.4 (5.0)	0.037
Moderate-to-severe exacerbations	10.5 (8.5)	13.7 (10.4)	−23.4 (4.8)	0.040
*ICS users (N = 135)*				
All exacerbations	9.3 (7.7)	12.5 (9.8)	−22.4 (5.9)	0.041
Mild exacerbations	7.9 (5.9)	10.1 (8.3)	−21.8 (5.2)	0.028
Moderate-to-severe exacerbations	10.9 (9.2)	14.0 (11.8)	−22.1 (4.8)	0.049
*ICS non-users (N = 119)*				
All exacerbations	9.0 (7.3)	12.1 (9.8)	−25.6 (5.4)	0.019
Mild exacerbations	7.8 (5.4)	9.7 (8.1)	−19.6 (5.1)	0.048
Moderate-to-severe exacerbations	10.7 (8.8)	13.9 (9.8)	−23.0 (5.0)	0.043

^a^ICS alone or combined with adrenergic agents; All RESTORE group: erdosteine (n = 165), placebo (n = 173); moderate COPD subgroup: erdosteine (n = 88), placebo (n = 91); ^b^*P*-value for comparison between erdosteine and placebo group from Wilcoxon rank-sum test (exacerbation rate) or log rank test (time to first exacerbation) or Mann-Whitney U-test (duration of exacerbation); ^c^Number of patients for which the time to first exacerbation is available. All comparisons between ICS users and non-users were non-significant; *ICS* inhaled corticosteroids

**Table 2:** Summary of ICS use data in pivotal studies of NAC, S-CMC and erdosteine in COPD

Study [Ref]	BRONCUS [99]	PANTHEON [93]	PANTHEON post-hoc [100]	PEACE [94]	RESTORE [95]	RESTORE post-hoc [101]
Mucolytic	N-acetylcysteine	N-acetylcysteine	N-acetylcysteine	Carbocysteine	Erdosteine	Erdosteine
Daily dose used	600 mg	1200 mg	1200 mg	1500 mg	600 mg	600 mg
Approved daily dose	600 mg	600 mg	600 mg	1500 mg	600 mg	600 mg
Study population	European	Chinese	Chinese	Chinese	European	European
Patients treated with ICS in the study, %	69.7	51.9	51.9	16.7	72.4	76.0
Efficacy of mucolytic irrespective of ICS use	NO	YES	NO	YES	YES	YES
Effect of mucolytic on exacerbation rate	Only in ICS non-users	YES	Only in ICS non-users	YES	YES	YES
Effect of mucolytic on exacerbation duration	No data	YES	No data	No data	YES	YES

*COPD* chronic obstructive pulmonary disease, *ICS* inhaled corticosteroid

A recent Cochrane systematic review with meta-analysis [102] concluded that there is no certainty about the existence of a specific population of patients with COPD that could benefit specifically from antioxidant treatment. However, this review does recognize that NAC, S-CMC and erdosteine can lead to a reduction in COPD exacerbation risk. Of the six clinical trials included in the systematic review considered to have a low risk of selection bias due to adequate allocation concealment, only the RESTORE trial reported a notable benefit of treatment in preventing exacerbations [102]. Clearly, it cannot be disregarded that the results of any meta-analysis refer to indirect comparisons between drugs in the Bayesian process, and thus the quality of evidence of any network meta-analysis is moderate [103]. Further limitations are the difference, or missing data, regarding the baseline characteristics of COPD patients enrolled in the clinical trials included in the meta-analysis, i.e. current smoking level, respiratory function impairment and COPD exacerbation rate in the previous year. Furthermore, the definition of COPD exacerbation was also inconsistent between the studies included in this meta-analysis [103]. Moreover, a study in COPD patients found that treatment with a high dose of NAC (3600 mg/day) had no clinical benefit including an improvement in respiratory health status, measured using the St George’s Respiratory Questionnaire [104]. Furthermore, this trial was terminated early due to safety concerns that administering very large dose of NAC to patients with severe COPD could stimulate tumour growth and proliferation.

Considering the clinical evidence collected over the years (and discussed above), the 2023 GOLD Report confirmed that NAC and S-CMC may reduce exacerbations in COPD patients not receiving ICS, whereas erdosteine has been shown to have a significant effect on exacerbations irrespective of the concurrent treatment with ICS [29].

## Conclusions

The question of whether antioxidants should be used instead of ICS or in combination with ICS in COPD patients is widely discussed. The latest GOLD Report confirmed that antioxidants like erdosteine, NAC and S-CMC can be effective as add-on therapy for the treatment of COPD patients. NAC and S-CMC can reduce the risk of exacerbations, but this effect is limited to patients not receiving ICS, while erdosteine has proven to be effective independently of ICS use, thus also in patients using ICS.

Although oxidative stress may play an important role in amplifying chronic lung inflammation in COPD, little is currently known about the involvement of oxidative stress in the different phenotypes, endotypes and etiotypes of COPD. Thus, the current recommendations on the use of thiol-based mucoactive drugs in COPD do not allow for personalised therapeutic management.

Nevertheless, some experts have recommended that long-term treatment with thiols should be used in patients with exacerbation phenotype without chronic bronchitis, particularly in those with early stages of COPD with less severe airway obstruction, bronchiectasis with COPD overlap phenotype, and bronchitic phenotype with or without history of exacerbation [105]. Combination with other phenotype-specific drugs in frequent exacerbators (ICS and even roflumilast) is possible and may be beneficial.

However, since it remains unclear whether patients with a bronchitis phenotype of COPD benefit more from mucolytic therapy than patients with other phenotypes [106], further studies should be undertaken to determine the place of thiols alone and in combination with an ICS in the optimal (and personalised) treatment of COPD.

## Data Availability

Not applicable.

## Abbreviations

AP-1: activated protein-1
BAL: bronchoalveolar lavage
COPD: chronic obstructive pulmonary disease
DAMP: danger-associated molecular patterns
EGFR: epidermal growth factor receptor
FEV_1_: forced expiratory volume in one second
GOLD: Global Initiative for Chronic Obstructive Lung Disease
GPX: glutathione peroxidase
GR: glucocorticoid receptor
GSH: glutathione
HDAC-2: histone deacetylase-2
ICS: inhaled corticosteroid
IL-8: interleukin-8
LABA: long-acting beta-agonist
LAMA: long-acting muscarinic antagonist
LPS: lipopolysaccharide
MAPK: mitogen-activated protein kinase
MDA: malondialdehyde
Met-1: metabolite 1
MPO: myeloperoxidase
NAC: N-acetyl-L-cysteine
NF-κB: nuclear factor-κB
NADPH: nicotinamide adenine dinucleotide phosphate
NOX: NADPH oxidases
PDGFR: platelet-derived growth factor receptor
PI3Kδ: phosphoinositide-3-kinase-δ
PKC: protein kinase C
ROS: reactive oxygen species
S-CMC: S-carboxymethylcysteine
SOD: superoxide dismutase
TEAC: Trolox equivalent antioxidant capacity
